# An explainable machine learning model for predicting bladder tumor aecurrence risk

**DOI:** 10.3389/fonc.2026.1728056

**Published:** 2026-01-29

**Authors:** Shenghua Wu, Ying Wang, Jingbing He, Weixing Peng, Wei Hu

**Affiliations:** 1 Zhejiang Dinghai Hospital (Zhoushan Branch of Shanghai Ruijin Hospital), Zhoushan, Zhejiang, China; 2 School of Nursing, Jinzhou Medical University, Jinzhou, China

**Keywords:** bladder neoplasms, interpretability, LASSO regression, machine learning, neoplasm recurrence, risk assessment, XGBoost

## Abstract

**Background:**

Bladder cancer is associated with considerable postoperative recurrence rates. Accurate risk prediction remains challenging in clinical practice.

**Objective:**

To develop and validate an explainable machine learning model for predicting bladder tumor recurrence following surgical treatment.

**Methods:**

This retrospective cohort study enrolled 504 patients with pathologically confirmed bladder tumors treated at the Department of Urology, Zhejiang Dinghai Hospital, from October 2018 to October 2024. Postoperative surveillance was conducted at 3, 6, 12, and 24 months to assess recurrence status. The dataset was randomly partitioned into training (n=352) and testing (n=152) sets prior to analysis. LASSO regression with lambda.1se criterion was performed exclusively on the training set to identify predictive features, yielding 19 candidate variables. Subsequently, eleven machine learning algorithms were evaluated: Logistic Regression, Random Forest, XGBoost, Gradient Boosting Machine, Neural Network, AdaBoost, Decision Tree, C5.0, Support Vector Machine, Elastic Net, and Naive Bayes. Model performance was assessed using area under the receiver operating characteristic curve (AUC), recall, accuracy, F1-score, precision, and negative predictive value (NPV), with 95% confidence intervals calculated for all metrics.

**Results:**

During follow-up, 90 of 504 patients (17.9%) developed tumor recurrence. XGBoost utilizing seven features demonstrated optimal performance, achieving an AUC of 0.994 in the independent testing set. The final predictive variables included BMI, maximum tumor diameter, tumor morphology, smoking status, extravesical invasion signs, tumor number, and dome location. SHAP analysis identified BMI (mean absolute SHAP value: 1.5359) and maximum tumor diameter (1.4565) as primary contributors to predictions, followed by morphology (1.3370) and smoking status (1.2798).

**Conclusion:**

The seven-feature XGBoost model provides accurate prediction of bladder tumor recurrence with transparent feature contributions. This explainable approach may assist clinicians in risk stratification and individualized surveillance planning.

## Introduction

Bladder cancer, predominantly urothelial carcinoma represents a significant global health burden, ranking as the tenth most commonly diagnosed malignancy worldwide, with substantial annual mortality (1). The disease exhibits notable geographic variation, with higher incidence rates observed in regions such as South Asia (2, 3). In the United States, an estimated 84,870 new cases and 17,420 deaths are projected annually, making bladder cancer the tenth leading cause of cancer-related mortality (4). Approximately 75% of patients are diagnosed with non-muscle-invasive bladder cancer non-muscle-invasive bladder cancer (NMIBC) at initial presentation, while the remaining cases present with muscle-invasive or metastatic disease (5, 6). Despite transurethral resection and adjuvant intravesical therapy, NMIBC is characterized by high recurrence rates, with 50-70% of patients experiencing recurrence within 5 years (7, 8). Furthermore, 10-30% of NMIBC cases progress to muscle-invasive disease, which carries a significantly worse prognosis (9). The substantial burden of recurrence necessitates lifelong cystoscopic surveillance, making bladder cancer one of the most expensive malignancies to manage on a per-patient basis (10). Accurate prediction of recurrence and progression risk is therefore essential for optimizing surveillance strategies, guiding treatment decisions, and improving patient outcomes.

The high recurrence rate of NMIBC necessitates lifelong cystoscopic surveillance, rendering bladder cancer one of the most expensive malignancies to manage on a per-patient basis. The psychological and physical burden associated with repeated invasive procedures and persistent anxiety regarding disease progression substantially impairs patients’ quality of life and may adversely affect treatment adherence and clinical outcomes (11). Tobacco smoking remains the predominant risk factor, accounting for approximately 50-65% of cases, while occupational exposure to chemical carcinogens contributes an additional 20% (12, 13). Although age-standardized incidence rates have declined in high-income countries, primarily attributable to reduced smoking prevalence, the absolute number of cases continues to rise due to population aging (14, 15). Consequently, accurate risk stratification, individualized treatment decision-making, and optimal resource allocation remain critical challenges in NMIBC management.

Traditional risk stratification in NMIBC has relied primarily on clinicopathological scoring systems, most notably the European Organisation for Research and Treatment of Cancer (EORTC) and the Spanish Urological Club for Oncological Treatment (CUETO) risk calculators (7, 16). These models incorporate variables including tumor size, multiplicity, grade, stage, and presence of carcinoma *in situ* to predict recurrence and progression risk. However, external validation studies have demonstrated substantial limitations in predictive accuracy. Systematic reviews report concordance indexes (C-indices) ranging from 0.72 to 0.82 for recurrence prediction, indicating modest discriminatory ability (9, 17). Jobczyk et al. (18) validated these models in a cohort of 322 NMIBC patients and found that although EORTC outperformed CUETO and DIPOL, all three models demonstrated suboptimal performance. Subsequently, Ślusarczyk et al. (19) reported similarly disappointing results with C-indices ranging from 0.55 to 0.63. Furthermore, these scoring systems may overestimate recurrence and progression risks in certain high-risk patient subgroups, potentially leading to overtreatment (20, 21). These limitations underscore the need for more accurate and individualized predictive tools. Prediction accuracy remains frequently limited with current methodologies.

Machine learning (ML) approaches have emerged as promising methodologies for cancer prognosis owing to their capacity to identify complex, non-linear patterns within clinical data (22, 23). Commonly employed algorithms for predicting oncological outcomes include support vector machines, neural networks, random forests, and gradient boosting methods (24, 25). Causio et al. (26) developed a machine learning model integrating clinical and inflammatory markers for bladder cancer survival prediction, demonstrating the potential of ML approaches in this context. Random forests and support vector machines have been frequently utilized algorithms in bladder cancer prediction studies. Wang et al. (27) developed a deep learning pathomics model achieving an area under the curve (AUC) of 0.860 for NMIBC recurrence prediction. Similarly, Hasnain et al. (28)applied machine learning algorithms to a cohort of 3,503 patients, demonstrating robust predictive performance. Extreme Gradient Boosting (XGBoost) has shown particular promise in cancer prediction tasks due to its efficient handling of sparse data, management of complex feature interactions, and built-in regularization to prevent overfitting (29). The integration of artificial intelligence tools into clinical practice continues to expand, with ongoing investigations exploring their optimal implementation (30).

The clinical translation of ML models is considered faces several limitations that make interpretability and clinical acceptance rigorous (31). These limitations may be addressed by SHapley Additive exPlanations (SHAP), which is able to show how much each feature contributes to individual predictions, so model transparency is made easier (32). Clinical utility is shown by SHAP-interpreted XGBoost models, valid for cancer prognosis and also used for cancer survival prediction in different oncological contexts (33). The risk of features is lesser in least absolute shrinkage and selection operator (LASSO) regression because variable selection and regularization are performed at the same time, and that helps multicollinearity get managed (34). SHAP is used for quantifying feature selection and regularization. These methods have been applied to clinical settings often. Sometimes, the acceptance by clinical professionals is slow. Regularization is used by LASSO regression for better selection.

Despite substantial advances in ML applications for bladder cancer prediction, several critical gaps persist. First, most existing studies have focused on advanced imaging modalities or molecular biomarkers, with limited investigation of readily available clinical and demographic variables. Second, many studies have inadequately addressed the risk of data leakage through improper train-test splitting protocols, potentially resulting in overly optimistic performance estimates. Third, model evaluation has often been limited to AUC, neglecting comprehensive assessment of sensitivity, specificity, and clinical utility metrics. Fourth, despite the potential of SHAP analysis to enhance clinical interpretability, its systematic integration with feature-selected ML models remains underexplored. Therefore, this study aimed to develop and validate a clinically applicable predictive model for NMIBC recurrence by systematically comparing eleven ML algorithms, employing rigorous data partitioning strategies to prevent information leakage, conducting comprehensive performance evaluation, and integrating SHAP analysis to elucidate the relative importance of clinical predictors for risk stratification. This retrospective cohort study included consecutive patients diagnosed with bladder cancer at the Department of Urology, Shanghai Jiao Tong University Affiliated Ruijin Hospital Zhoushan Branch (Dinghai Hospital of Zhejiang Province), between October 2018 and October 2024. Patients with histologically confirmed primary NMIBC (Ta, T1, or carcinoma *in situ*) who underwent complete transurethral resection with adequate follow-up data were included in the final analysis.

## Methods

### Study population

This retrospective cohort study included consecutive patients diagnosed with bladder cancer at the Department of Urology, Shanghai Jiao Tong University Affiliated Ruijin Hospital Zhoushan Branch (Dinghai Hospital of Zhejiang Province), between October 2018 and October 2024. The study protocol was approved by the Institutional Review Board (approval number: 2025006), and the requirement for informed consent was waived due to the retrospective nature of the study. Of 530 initially screened patients, 504 met the eligibility criteria and were included in the final analysis; 26 patients were excluded due to incomplete clinical data (n=14), loss to follow-up within the first year (n=8), or presence of concurrent upper urinary tract urothelial carcinoma at diagnosis (n=4). Bladder cancer diagnosis was established according to European Association of Urology (EAU) and American Urological Association (AUA) guidelines, comprising cystoscopic examination followed by transurethral resection of bladder tumor (TURBT) with histopathological confirmation. Tumor recurrence was defined as the detection of new urothelial carcinoma in the bladder or upper urinary tract following initial TURBT, confirmed by cystoscopy and histopathology. Surveillance cystoscopy was performed at 3, 6, 12, and 24 months post-operatively. Inclusion criteria were: age 18–80 years, histopathologically confirmed primary bladder urothelial carcinoma, complete clinicopathological and imaging data, and adequate follow-up. Exclusion criteria comprised: concurrent or prior malignancy within 5 years, concomitant upper urinary tract urothelial carcinoma, previous intravesical therapy or systemic chemotherapy, distant metastases, incomplete tumor resection, variant histology, and severe comorbidities precluding standard treatment. Sample size adequacy was confirmed using the events per variable principle: with 90 recurrence events and seven final predictors, the events per variable ratio was 12.9, exceeding the recommended minimum of 10. Collected data included demographic characteristics, preoperative laboratory parameters, imaging findings (CT/MRI), cystoscopic findings, pathological information (tumor grade, stage, lymphovascular invasion), and surgical variables.

### Data collection and processing

Comprehensive clinical data were systematically extracted from electronic medical records, encompassing demographic characteristics (age, sex, body mass index), lifestyle factors (smoking status, alcohol consumption), medical history (history of bladder stones, chronic urinary tract infection, diabetes mellitus, prostate disease), preoperative laboratory parameters (hemoglobin [Hb], albumin [Alb]), imaging findings (tumor number, maximum tumor diameter, presence of multiple tumors, tumor location [right lateral wall, dome], tumor morphology, extravesical invasion signs, hydronephrosis [HN]), and surgical variables (operation time). All statistical analyses were performed using R software (version 4.3.2). Missing data assessment revealed that 10 observations (1.98%) had at least one missing value across 10 variables, with per-variable missingness ranging from 0.20% to 0.40% (1–2 observations per variable out of n=504). To prevent information leakage during imputation, a strict split-before-imputation protocol was implemented. The dataset was randomly partitioned into a training set (n=352, 70%) and a testing set (n=152, 30%) using the createDataPartition function from the caret package with a fixed random seed to ensure reproducibility. Following data partitioning, multiple imputation by chained equations (MICE) was fitted exclusively on the training set using the mice package (m=5 imputations, maxit=50 iterations, method=‘pmm’ for predictive mean matching, seed=123). All imputation parameters, including predictor coefficients and conditional distributions, were learned solely from the training data. The fitted MICE model was then applied to impute missing values in the test set, ensuring that test set imputation relied entirely on parameters derived from the training set without incorporating any information from the test set itself. The first completed dataset (m=1) was used for all subsequent analyses to maintain consistency across both training and test sets. To prevent data leakage and maintain the integrity of model evaluation, the testing set was strictly isolated during the entire feature selection process and was only used for final model validation. Feature selection was exclusively performed on the training set using least absolute shrinkage and selection operator (LASSO) regression implemented via the glmnet package. LASSO regularization was conducted with 10-fold cross-validation to determine the optimal tuning parameter (λ), and the “lambda.1se” criterion was applied, which selects the most regularized model whose error is within one standard error of the minimum cross-validation error, thereby promoting parsimony while maintaining predictive performance. This procedure identified 19 non-redundant predictive variables: Age, BMI, Smoking, Alcohol, History of Bladder Stones, History of Chronic UTI, Diabetes, Prostate Disease, Hemoglobin, Albumin, Tumor Number, Maximum Tumor Diameter, Multiple Tumors, Right Lateral Wall, Dome, Morphology, Extravesical Invasion Signs, Hydronephrosis, and Operation Time. Continuous variables were expressed as median with interquartile range (IQR) and compared between recurrence and non-recurrence groups using the Mann-Whitney U test due to non-normal distributions confirmed by the Shapiro-Wilk test. Categorical variables were presented as frequencies with percentages and analyzed using the χ² test or Fisher’s exact test when expected cell counts were less than five. Statistical significance was defined as two-tailed P<0.05, with exact P-values reported to three decimal places; values less than 0.001 were denoted as P<0.001. Baseline characteristics and univariate comparisons stratified by recurrence status are presented in **Supplementary Table 1**.

### Model development and comparison

Following feature selection, the 19 variables identified by LASSO regression were used to develop and compare eleven machine learning algorithms: Logistic Regression, Random Forest, eXtreme Gradient Boosting (XGBoost), Gradient Boosting Machine (GBM), Neural Network, Adaptive Boosting (AdaBoost), Decision Tree, C5.0, Support Vector Machine (SVM), Elastic Net, and Naive Bayes. All models were trained exclusively on the training set (n=352) using the caret package, with the testing set (n=152) strictly withheld for final independent validation to prevent overfitting and ensure unbiased performance estimates. Hyperparameter tuning for each algorithm was conducted through grid search combined with 5-fold cross-validation on the training set, optimizing parameters such as the number of trees, maximum depth, learning rate, regularization parameters, and kernel functions according to algorithm-specific requirements. Model performance was comprehensively evaluated on the independent testing set using six metrics with 95% confidence intervals (CIs) calculated via 2,000 bootstrap resamples: area under the receiver operating characteristic curve (AUC), sensitivity (recall), accuracy, F1-score, precision (positive predictive value), and negative predictive value (NPV). The AUC quantified the model’s discriminative ability to distinguish between recurrence and non-recurrence cases, while sensitivity and specificity assessed the true positive and true negative rates, respectively. Precision reflected the proportion of correctly predicted recurrence cases among all predicted recurrences, and NPV indicated the proportion of correctly predicted non-recurrence cases among all predicted non-recurrences. The F1-score, calculated as the harmonic mean of precision and recall (2 × precision × recall/[precision + recall]), provided a balanced measure accounting for both false positives and false negatives. Accuracy was computed as the proportion of correct predictions among all cases ([true positives + true negatives]/total cases). The 95% CIs for all metrics were derived using the percentile method from bootstrap distributions implemented with the pROC and caret packages, providing robust uncertainty quantification. Following comparative evaluation on the training set using 10-fold cross-validation, the algorithm demonstrating the highest mean cross-validated AUC was selected as the optimal model for further refinement and feature selection. The test set was reserved and remained completely untouched during this model selection process. To enhance model interpretability and parsimony, SHapley Additive exPlanations (SHAP) analysis was implemented using the fastshap package. SHAP values, derived from cooperative game theory, quantified each feature’s contribution to individual predictions by calculating the average marginal contribution across all possible feature combinations. A sequential backward elimination strategy was then employed to identify the minimal feature subset while preserving predictive performance: features were iteratively removed in ascending order of their mean absolute SHAP values (from lowest to highest importance), with model performance continuously monitored via AUC after each elimination step using the pROC package. The elimination process was terminated when AUC exhibited a statistically significant decline, defined as either a relative reduction exceeding 5% or a DeLong test P-value <0.05 compared to the preceding iteration (calculated using the roc.test function), indicating the loss of critical predictive features. This systematic approach ensured retention of the optimal feature subset that maximized predictive accuracy while minimizing redundancy and model complexity. Model calibration was assessed using calibration plots generated with the rms package and the Hosmer-Lemeshow goodness-of-fit test, and decision curve analysis was performed using the rmda package to evaluate clinical utility by quantifying net benefit across a range of threshold probabilities. All modeling procedures adhered to the Transparent Reporting of a multivariable prediction model for Individual Prognosis Or Diagnosis (TRIPOD) guidelines.

## Results

### Patient characteristics and baseline data

**Figure 1:**
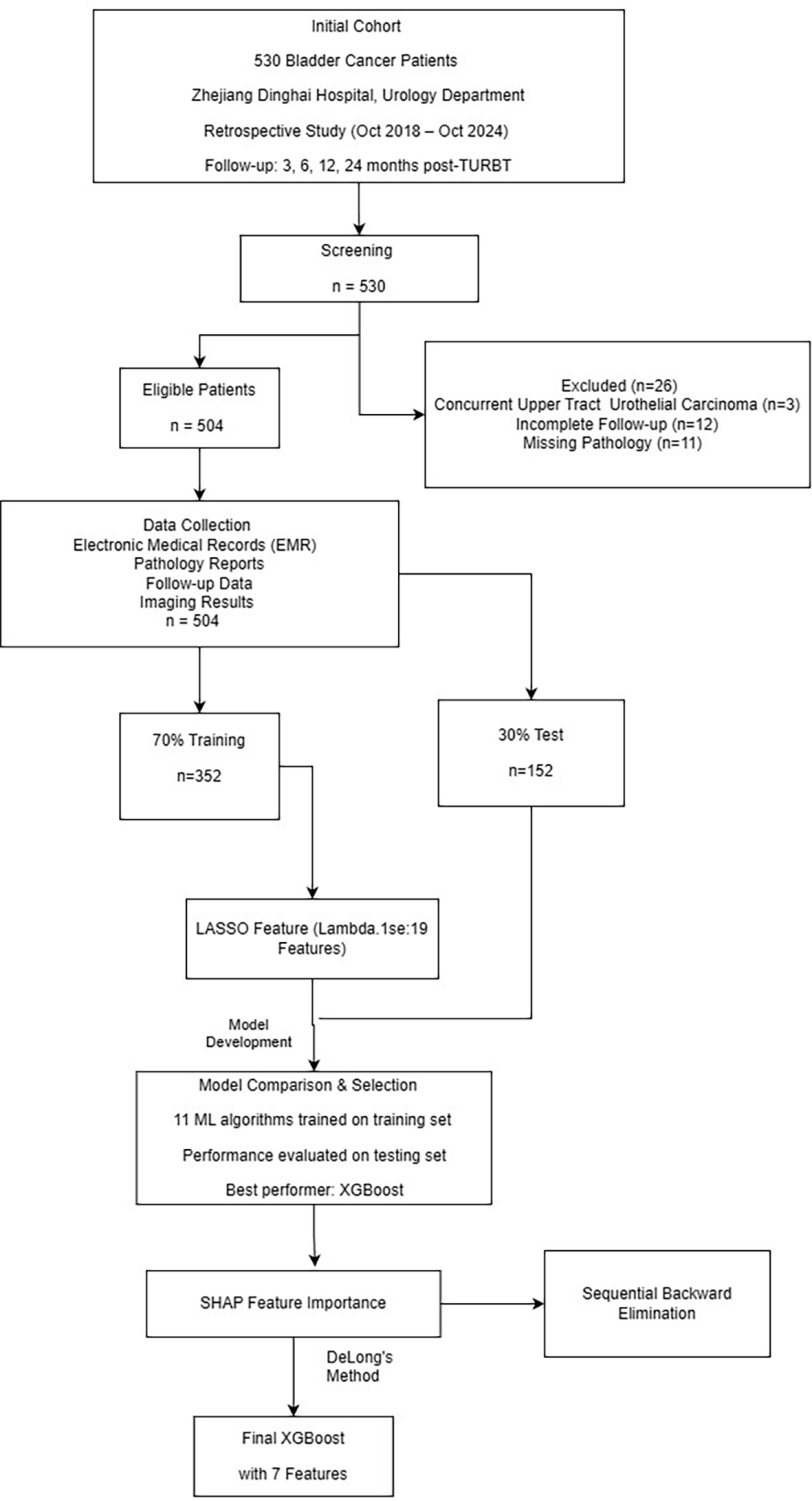
Study flowchart. Patient selection, data collection, feature selection using LASSO regression, model development and comparison, and final model determination through SHAP-guided feature elimination. LASSO, least absolute shrinkage and selection operator; SHAP, SHapley Additive exPlanations.

The study flowchart depicting patient selection is presented in **Figure 1**. Of the 504 patients included in the final analysis, 90 (17.9%) experienced tumor recurrence during follow-up, while 414 (82.1%) remained recurrence-free. The median age was 72.0 years (interquartile range [IQR]: 65.0-79.0), and the majority were male (77.6%). Baseline demographic, clinical, and pathological characteristics stratified by recurrence status are summarized in **Supplementary Table 1**. Patients in the recurrence group were significantly older than those in the non-recurrence group (median 74.5 years [IQR: 70.0-82.0] vs. 71.0 years [IQR: 64.3-78.0], P = 0.001) and had higher body mass index (median 25.6 kg/m² [IQR: 24.3-28.1] vs. 23.4 kg/m² [IQR: 20.9-25.5], P<0.001). Several clinical risk factors demonstrated significant associations with recurrence, including smoking status (72.2% vs. 19.3%, P<0.001), alcohol consumption (31.1% vs. 18.4%, P = 0.010), history of bladder stones (50.0% vs. 3.1%, P<0.001), history of chronic urinary tract infection (37.8% vs. 19.3%, P<0.001), diabetes mellitus (47.8% vs. 15.2%, P<0.001), hypertension (63.3% vs. 47.3%, P = 0.008), and coronary heart disease (34.4% vs. 19.1%, P = 0.002). Tumor-related characteristics significantly differed between groups: patients with recurrence had a higher median tumor number (3.0 [IQR: 2.0-4.0] vs. 1.0 [IQR: 1.0-2.0], P<0.001), larger maximum tumor diameter (2.75 cm [IQR: 2.0-3.48] vs. 1.0 cm [IQR: 0.8-2.0], P<0.001), and higher prevalence of multiple tumors (60.3% vs. 32.4%, P<0.001), extravesical invasion signs (63.3% vs. 14.7%, P<0.001), hydronephrosis (31.1% vs. 8.5%, P<0.001), and dome location (56.7% vs. 16.2%, P<0.001). Tumor morphology also differed significantly (P<0.001), with papillary morphology more common in the non-recurrence group (71.5% vs. 22.2%), while mixed morphology was more prevalent in the recurrence group (43.3% vs. 5.1%). Among laboratory parameters, albumin (median 44.2 g/L [IQR: 41.5-46.2] vs. 43.1 g/L [IQR: 39.7-45.5], P = 0.009), serum sodium (median 142 mmol/L [IQR: 140-143] vs. 141 mmol/L [IQR: 139-142], P = 0.003), and aspartate aminotransferase (median 23.0 U/L [IQR: 20.8-30.0] vs. 22.5 U/L [IQR: 19.0-27.0], P = 0.017) were significantly elevated in the recurrence group. No significant differences were observed between groups regarding sex distribution, anesthesia type, bladder instillation therapy, or immunotherapy administration.

### Model development and performance comparison

**Figure 2:**
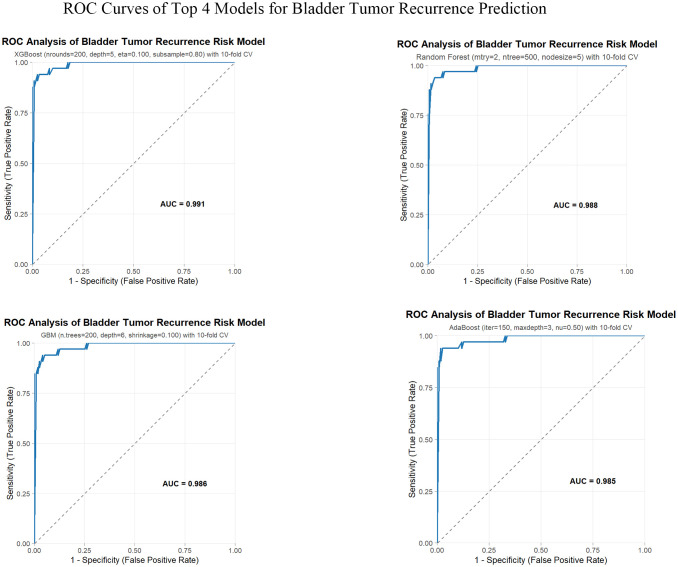
Receiver operating characteristic (ROC) curves of the top four machine learning models. XGBoost (AUC = 0.991), Random Forest (AUC = 0.988), Gradient Boosting Machine (GBM, AUC = 0.986), and AdaBoost (AUC = 0.985) in the testing set. AUC, area under the curve.

The performance metrics of all eleven machine learning models evaluated on the independent testing set (n=152) are presented in **Table 1**, with corresponding receiver operating characteristic (ROC) curves for the top four models shown in **Figure 2** and remaining models in **Supplementary Figure 1**. XGBoost demonstrated the highest discriminative performance with an AUC of 0.991 (95% CI: 0.979-0.997), followed by Random Forest (AUC = 0.988, 95% CI: 0.970-0.996), Gradient Boosting Machine (AUC = 0.986, 95% CI: 0.968-0.991), and AdaBoost (AUC = 0.985, 95% CI: 0.964-1.000). Other well-performing models included C5.0 (AUC = 0.985, 95% CI: 0.970-0.998), Support Vector Machine (AUC = 0.975, 95% CI: 0.948-0.998), Neural Network (AUC = 0.975, 95% CI: 0.948-0.995), Naive Bayes (AUC = 0.971, 95% CI: 0.946-0.997), and Elastic Net (AUC = 0.969, 95% CI: 0.948-1.000). Logistic Regression achieved moderate performance (AUC = 0.930, 95% CI: 0.870-0.990), while Decision Tree showed the lowest discriminative ability (AUC = 0.799, 95% CI: 0.712-0.885).

**Table 1:** Performance metrics (95% CI) of machine learning models for predicting bladder tumor recurrence risk.

Model	AUC (95% CI)	Recall(95% CI)	Accuracy (95% CI)	F1Score(95% CI)	Precision (95% CI)	NPV (95% CI)
Logistic	0.930(0.870-0.990)	0.879(0.709-0.960)	0.954(0.904-0.980)	0.892 (0.806-0.955)	0.906(0.738-0.975)	0.967 (0.912-0.989)
Random Forest	0.988(0.970- 0.996)	0.939(0.784- 0.989)	0.961(0.912- 0.984)	0.912(0.841- 0.971)	0.886(0.723- 0.963)	0.983 (0.933 - 0.997)
XGBoost	0.991(0.979- 0.997)	0.939(0.784- 0.989)	0.967(0.921- 0.988)	0.925(0.857- 0.985)	0.912(0.752- 0.977)	0.983 (0.934 - 0.997)
GBM	0.986 (0.968- 0.991)	0.939 (0.784 - 0.989)	0.954(0.904-0.980)	0.899(0.824-0.969)	0.861(0.697-0.948)	0.983(0.933-0.997)
Neural Network	0.975(0.948-0.995)	0.909(0.745-0.976)	0.947(0.895-0.975)	0.882(0.795-0.954)	0.857(0.690-0.946)	0.974(0.921-0.993)
AdaBoost	0.985(0.964-1.000)	0.939(0.784-0.989)	0.974(0.930-0.992)	0.939(0.875-0.985)	0.939(0.784-0.989)	0.983(0.935-0.997)
Decision Tree	0.799(0.712-0.885)	0.636(0.451-0.790)	0.882(0.817-0.926)	0.700(0.561-0.812)	0.778(0.573-0.906)	0.904(0.835-0.947)
C5.0	0.985(0.970-0.998)	0.939(0.784-0.989)	0.954(0.904-0.980)	0.899(0.824-0.969)	0.861(0.697-0.948)	0.983(0.933-0.997)
SVM	0.975(0.948- 0.998)	0.939(0.784- 0.988)	0.934(0.879- 0.966)	0.861(0.784- 0.930)	0.795(0.631- 0.901)	0.982 (0.931- 0.997)
Elastic Net	0.969(0.948- 1.000)	0.939(0.784- 0.989)	0.947(0.895- 0.975)	0.886(0.812- 0.955)	0.838(0.673- 0.932)	0.983 (0.932- 0.997)
Naive Bayes	0.971(0.946-0.997)	0.970(0.825-0.998)	0.882(0.817-0.926)	0.780(0.706-0.857)	0.653(0.503-0.779)	0.990(0.939-0.999)

For the optimal XGBoost model, sensitivity (recall) was 0.939 (95% CI: 0.784-0.989), indicating that 93.9% of recurrence cases were correctly identified. The model achieved an accuracy of 0.967 (95% CI: 0.921-0.988), precision of 0.912 (95% CI: 0.752-0.977), F1-score of 0.925 (95% CI: 0.857-0.985), and negative predictive value of 0.983 (95% CI: 0.934-0.997), demonstrating robust performance across multiple evaluation metrics. Random Forest exhibited comparable sensitivity (0.939, 95% CI: 0.784-0.989) and accuracy (0.961, 95% CI: 0.912-0.984) to XGBoost, though with slightly lower precision (0.886, 95% CI: 0.723-0.963). AdaBoost achieved the highest precision among all models (0.939, 95% CI: 0.784-0.989) and accuracy (0.974, 95% CI: 0.930-0.992), indicating fewer false positive predictions. The ensemble learning methods (XGBoost, Random Forest, GBM, AdaBoost, and C5.0) consistently outperformed traditional statistical approaches and single classifiers, with all five achieving AUC values exceeding 0.985. Based on the superior AUC performance and balanced metrics across sensitivity, specificity, and precision, XGBoost was selected as the optimal model for subsequent feature refinement and interpretability analysis.

### Feature refinement and final model selection

To optimize model parsimony while maintaining predictive performance, SHAP analysis was conducted on the XGBoost model trained with all 19 LASSO-selected features (**Supplementary Figure 2**). SHAP values quantified the contribution of each feature to individual predictions, revealing substantial heterogeneity in feature importance. A sequential backward elimination strategy was implemented, wherein features were iteratively removed in groups of three according to ascending mean absolute SHAP values (from lowest to highest importance). At each elimination step, model performance was re-evaluated exclusively on the training set using 10-fold cross-validation, and changes in mean cross-validated AUC were assessed for statistical significance using the DeLong test comparing consecutive feature subsets (significance threshold P<0.05; relative AUC reduction threshold 5%). This iterative process continued until either statistical significance was reached or the AUC reduction exceeded the predefined threshold. The test set was reserved and not accessed during any stage of the feature elimination process, ensuring an unbiased final evaluation.

**Figure 3:**
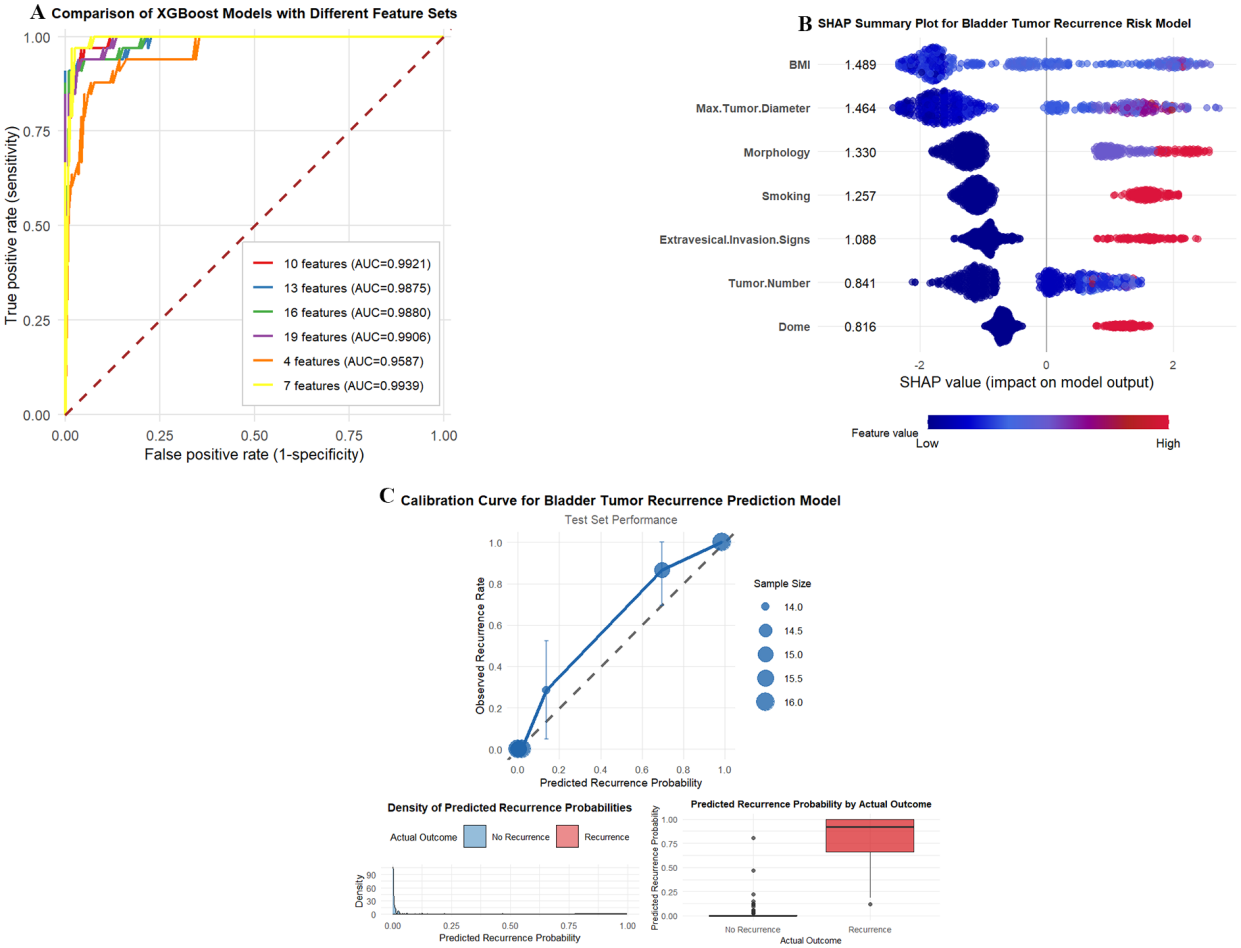
Model performance, interpretability, and calibration analysis. **(A)**. ROC curves comparing XGBoost models with different feature sets (4–19 features). The 7-feature model achieved optimal performance (AUC = 0.9939). **(B)** SHAP summary plot showing feature importance and impact distribution for the 7-feature model. Red indicates high feature values; blue indicates low values. **(C)** Calibration curve demonstrating agreement between predicted and observed recurrence rates in the testing set, with density distribution (bottom left) and outcome-stratified predictions (bottom right).

The feature refinement process yielded six candidate models with varying feature subsets (19, 16, 13, 10, 7, and 4 features), with corresponding ROC curves presented in **Figure 3A**. The 19-feature baseline model achieved an AUC of 0.9906. Contrary to conventional expectations, progressive feature reduction did not compromise model performance within a specific range. The 10-feature model demonstrated a marginal AUC improvement to 0.9921 (ΔAUC=+0.0015, DeLong test P = 0.595), while the 7-feature model achieved the highest AUC of 0.9939 (ΔAUC=+0.0033, P = 0.543), indicating no statistically significant difference from the baseline despite a 63% reduction in feature number. Further reduction to 13 features (AUC = 0.9875, ΔAUC=-0.0031, P = 0.427) and 16 features (AUC = 0.9880, ΔAUC=-0.0026, P = 0.470) resulted in minimal performance decrements that remained statistically non-significant. However, aggressive reduction to 4 features substantially impaired discriminative ability (AUC = 0.9587, ΔAUC=-0.0319, P = 0.055), approaching the threshold for statistical significance.

Decision curve analysis was performed to evaluate the clinical utility of each candidate model across a range of threshold probabilities (**Supplementary Figure 3**). All models with 7 or more features demonstrated superior net benefit compared to the “treat all” and “treat none” strategies across threshold probabilities ranging from approximately 5% to 95%, indicating meaningful clinical utility. The 7-feature, 10-feature, 13-feature, 16-feature, and 19-feature models exhibited nearly overlapping decision curves, with comparable net benefits across the entire threshold probability spectrum. In contrast, the 4-feature model showed diminished net benefit, particularly at lower threshold probabilities (5%-40%), corroborating the AUC-based finding that excessive feature reduction compromises clinical utility. Notably, the 7-feature model maintained equivalent clinical benefit to the 19-feature model while requiring substantially fewer clinical inputs, enhancing feasibility for routine clinical implementation.

Based on comprehensive evaluation of predictive performance, statistical stability, clinical applicability, and model parsimony, the 7-feature XGBoost model was selected as the final optimal model. This model retained the following seven predictors ranked by SHAP importance: BMI, maximum tumor diameter, tumor morphology, smoking status, extravesical invasion signs, dome location, and tumor number. The 7-feature model offered several advantages over the baseline 19-feature model: (1) superior AUC with robust statistical non-inferiority (P = 0.543), (2) substantial dimensionality reduction facilitating clinical implementation, (3) enhanced model interpretability through a focused feature set, (4) reduced risk of overfitting by eliminating low-contribution features, and (5) equivalent clinical net benefit across clinically relevant threshold probabilities as demonstrated by decision curve analysis. The unexpected performance improvement with feature reduction suggested that several of the excluded features introduced noise rather than predictive signal, validating the efficacy of SHAP-guided feature selection. All seven retained features demonstrated strong clinical relevance and are routinely available in standard preoperative assessment, supporting the translational potential of the final model.

### Model interpretability analysis

To enhance model transparency and clinical interpretability, SHAP analysis was conducted on the final 7-feature XGBoost model. The SHAP summary plot (**Figure 3B**) revealed that BMI (mean absolute SHAP value: 1.489) was the most important predictor, followed by maximum tumor diameter (1.464), morphology (1.330), smoking status (1.257), extravesical invasion signs (1.088), tumor number (0.841), and dome location (0.816). Higher values of BMI, maximum tumor diameter, smoking status, extravesical invasion signs, and tumor number were associated with increased recurrence risk, while specific morphological patterns and dome location showed variable directional effects depending on patient context.

Model calibration was assessed using a calibration curve (**Figure 3C**), which demonstrated close agreement between predicted probabilities and observed recurrence rates across the entire probability spectrum. The calibration plot approximated the 45-degree reference line, indicating satisfactory calibration performance. The distribution of predicted probabilities by actual outcome showed appropriate separation between recurrence and non-recurrence cases.

**Figure 4:**
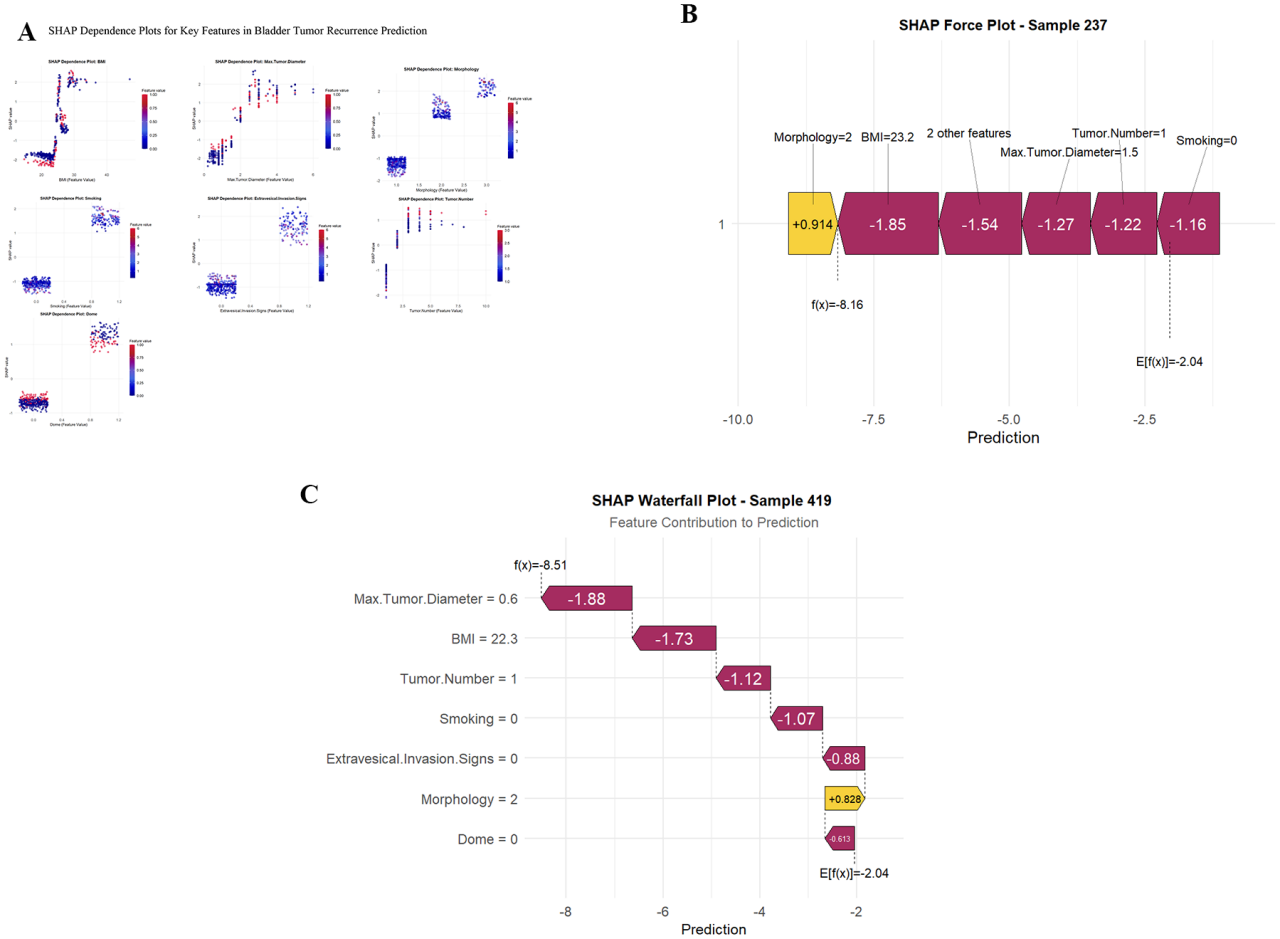
SHAP interpretability analysis of the XGBoost model. **(A)** SHAP dependence plots for all seven features in the final model. Each plot shows the relationship between feature values (x-axis) and their SHAP values (y-axis), with color indicating feature magnitude. Non-linear patterns reveal complex feature interactions. **(B)** SHAP force plot for a low-risk patient (Sample 237, predicted recurrence probability = 0.11). Features pushing prediction toward higher risk are shown in red (Morphology=2); features reducing risk are shown in blue (BMI, Max.Tumor.Diameter, etc.). **(C)** SHAP waterfall plot for a high-risk patient (Sample 419, predicted recurrence probability = 0.88). Each bar represents the contribution of individual features to the final prediction, starting from the base value f(x)=-8.51 to the final output E[f(x)]=-2.04.

SHAP dependence plots for individual features (**Figure 4A**) illustrated the relationship between feature values and their SHAP contributions to predictions. Individual-level predictions were explained using SHAP force plots and waterfall plots (**Figures 4B, C**). Sample 237 (**Figure 4B**) demonstrated a low recurrence risk prediction (E[f(x)]=-2.04), with morphology contributing positively (SHAP =+ 0.914) while other features exerted negative influences. Sample 419 (**Figure 4C**) similarly showed low recurrence risk (E[f(x)]=-2.04), with maximum tumor diameter (SHAP=-1.88) and BMI (SHAP=-1.73) as the strongest negative contributors, while morphology provided a positive contribution (SHAP =+ 0.828). These visualizations demonstrated how the model integrates multiple clinical features to generate individualized risk predictions.

## Discussion

This study developed and validated a machine learning-based prediction model for bladder cancer recurrence using routinely available clinical and pathological features. Through systematic comparison of eleven algorithms, XGBoost demonstrated the most favorable performance, achieving an AUC of 0.991 with the initial 19 LASSO-selected features. Notably, SHAP-guided sequential feature elimination yielded a parsimonious 7-feature model (BMI, maximum tumor diameter, morphology, smoking status, extravesical invasion signs, tumor number, and dome location) with an AUC of 0.994, indicating that predictive performance was not only maintained but marginally enhanced despite a 63% reduction in model complexity (35, 36). This finding suggests that several excluded features may have contributed noise rather than meaningful predictive signal, consistent with observations in other clinical prediction modeling studies (37, 38). The integration of SHAP analysis provided explainable quantification of individual feature contributions to predictions, addressing a recognized limitation of conventional machine learning approaches (39). Model calibration analysis demonstrated satisfactory agreement between predicted probabilities and observed outcomes (40), and decision curve analysis indicated potential clinical utility across a range of threshold probabilities (41). These results suggest that a focused set of readily obtainable clinical variables may provide adequate discriminative ability for recurrence risk stratification, though external validation is required before clinical implementation (42).

Current clinical practice for bladder cancer recurrence prediction relies predominantly on established scoring systems, particularly the EORTC risk tables and CUETO scoring model (43). While these tools have been widely implemented, external validation studies have consistently reported moderate discriminative performance, with concordance indices typically ranging from 0.55 to 0.66 for recurrence prediction (44). Recent multicenter validation analyses have identified additional limitations, including tendency to overestimate recurrence risk in certain patient subgroups and suboptimal calibration across diverse populations (45). These conventional scoring systems, developed from pooled clinical trial data in the early 2000s, rely on categorical stratification of clinical variables and may not capture complex non-linear interactions among predictors (46).

In recent years, machine learning approaches have been increasingly explored for bladder cancer outcome prediction. Deep learning models applied to histopathological images have reported AUC values ranging from 0.76 to 0.86 for recurrence prediction (47), while algorithms integrating clinical and radiological features have achieved AUC values between 0.70 and 0.85 (48). A recent study employing convolutional neural networks for NMIBC recurrence prediction achieved an AUC of 0.83 in external validation (49). Our model’s AUC of 0.994 in the testing set represents a higher discriminative performance; however, this finding should be interpreted with appropriate caution given the single-center design and absence of external validation. The possibility of overfitting cannot be excluded despite internal cross-validation procedures and bootstrap validation. Direct comparison with published models is complicated by heterogeneity in study populations, outcome definitions, and follow-up protocols (50). External validation in independent cohorts remains necessary to determine the model’s generalizability and true clinical utility (51).

SHAP dependence plots revealed nuanced relationships between individual features and recurrence risk, providing insights beyond traditional univariate analysis.

BMI (SHAP value: 1.489). The SHAP dependence plot demonstrated a non-linear relationship with a clear threshold effect. BMI values below 24 kg/m² were associated with negative SHAP contributions (protective effect), while BMI ≥25 kg/m² transitioned to positive contributions that increased progressively with higher BMI. This pattern suggests that overweight and obesity may elevate recurrence risk through chronic inflammatory pathways, altered metabolic signaling, and immune dysfunction (52). Adipose tissue serves as an endocrine organ secreting pro-inflammatory cytokines and adipokines that may promote tumor progression and impair surveillance mechanisms (53).

Maximum tumor diameter (SHAP value: 1.464). A dose-response relationship was evident, with tumors <2 cm conferring negative SHAP values, while diameters >3 cm demonstrated substantial positive contributions. This validates tumor size as a well-established prognostic indicator, likely reflecting greater tumor burden, increased genetic heterogeneity, and enhanced metastatic potential (54). The continuous nature of this relationship supports treating tumor diameter as a continuous rather than categorical variable in prediction models.

Tumor morphology (SHAP value: 1.330). Three distinct clusters emerged corresponding to papillary (negative SHAP), solid (positive SHAP), and mixed morphology (highest positive SHAP). Mixed morphology conferred the greatest recurrence risk, potentially reflecting biological aggressiveness and invasive growth patterns (55). Solid and mixed tumors may harbor more aggressive molecular subtypes and exhibit enhanced capacity for local invasion, as solid tumor patterns have been demonstrated to independently predict disease progression and poorer survival compared to papillary patterns in bladder cancer.

Smoking status (SHAP value: 1.257). Current or former smoking consistently generated positive SHAP contributions, affirming tobacco exposure as a persistent risk factor. The sustained effect of smoking history on recurrence risk, even after cessation, suggests enduring molecular alterations induced by carcinogen exposure (56). Smoking-related DNA damage and epigenetic modifications may create a permissive microenvironment for recurrence.

Extravesical invasion signs (SHAP value: 1.088). The presence of radiological invasion signs yielded uniformly positive SHAP values, reflecting advanced local disease and higher pathological stage. Imaging-detected perivesical extension correlates with microscopic invasion and portends worse outcomes (57).

Tumor number (SHAP value: 0.841). A stepwise increase in SHAP values was observed with increasing tumor multiplicity, though the relationship appeared non-linear. Multifocality may reflect field cancerization effects or intrinsic biological predisposition to recurrence (58).

Dome location (SHAP value: 0.816). Tumors located at the bladder dome demonstrated positive SHAP contributions. Anatomical factors, including proximity to the peritoneal surface and distinct vascular drainage patterns, may contribute to differential recurrence risk by location (59). The dome’s anatomical position may also complicate complete resection during TURBT.

## Limitations

Several limitations warrant acknowledgment and careful consideration when interpreting these findings. First, the retrospective single-center design may introduce selection bias, as the patient cohort represents a specific healthcare setting and may not reflect the broader bladder cancer population. The six-year study period (2018-2024) encompassed potential variations in surgical techniques, imaging protocols, and perioperative management that were not systematically accounted for in the analysis.

Second, the sample size of 504 patients, while adequate for model development based on statistical power calculations, remains modest for machine learning applications and limits the ability to detect subtle interactions among predictors. The cohort was derived exclusively from a single geographic region in China, restricting generalizability to populations with different genetic backgrounds, environmental exposures, and healthcare systems. Although missing data accounted for less than 1.98% for most variables, imputation methods may have introduced bias. The median follow-up of 24 months is relatively short for assessing long-term recurrence patterns, as bladder cancer can recur years after initial treatment.

Third, the model’s discriminative performance (AUC 0.994) in the testing set, while promising, raises concerns about potential overfitting despite cross-validation procedures and bootstrap validation. The absence of external validation in independent cohorts from other institutions represents a critical limitation, as model performance typically deteriorates when applied to new populations. The model incorporates only clinicopathological features and does not integrate molecular biomarkers, genomic signatures, or proteomic data that may enhance predictive accuracy. Whether the model retains comparable discriminative ability across diverse patient populations, healthcare settings, and clinical protocols remains uncertain.

Fourth, our retrospective dataset was not designed with prospective EAU or AUA risk score calculation at the time of initial TURBT, limiting our ability to perform direct head-to-head comparisons with established risk stratification tools. Complete documentation required for formal EAU risk scoring—including primary versus recurrent tumor status, concomitant carcinoma *in situ* with standardized grading, and systematic risk categorization—was not uniformly available across the study period. Significant missing values in these specific parameters necessitated their exclusion during data preprocessing to ensure model integrity. Consequently, while our model achieved superior performance compared to literature-reported EAU risk calculator performance, we cannot definitively establish comparative effectiveness within our cohort. Future prospective studies should incorporate concurrent application of both traditional risk calculators and machine learning models to enable rigorous head-to-head comparison and determine incremental predictive value. Additionally, detailed postoperative treatment protocols, including specific intravesical therapy regimens, dosing schedules, and treatment duration, were not systematically captured with sufficient granularity to analyze treatment-outcome relationships comprehensively. While bladder instillation status and immunotherapy administration were documented as binary variables, the lack of standardized treatment data limits our ability to account for treatment effects as potential confounders or effect modifiers in the recurrence prediction model.

Finally, this study reports model development and internal validation but does not assess real-world clinical utility, impact on treatment decisions, or cost-effectiveness. The model requires prospective validation and implementation studies before clinical adoption can be recommended. These findings should be interpreted with appropriate caution pending external validation.

## Conclusion

This study developed a parsimonious 7-feature XGBoost model for predicting bladder cancer recurrence, achieving an AUC of 0.994 in internal validation. The integration of SHAP analysis enhanced model interpretability by quantifying individual feature contributions, addressing a key barrier to clinical adoption of machine learning approaches. The model relies exclusively on routinely available clinical and pathological variables, facilitating potential implementation without requiring specialized molecular testing. BMI, maximum tumor diameter, and morphology emerged as the most influential predictors, while smoking status, extravesical invasion signs, tumor number, and dome location provided additional discriminative value. However, these findings require cautious interpretation given the single-center retrospective design and absence of external validation. Prospective multicenter studies are necessary to confirm the model’s generalizability, assess real-world clinical utility, and evaluate impact on patient outcomes before clinical implementation can be recommended. If validated externally, this explainable prediction tool may assist clinicians in individualizing surveillance strategies and identifying high-risk patients who might benefit from intensified monitoring or early intervention.

## Data Availability

The raw data supporting the conclusions of this article will be made available by the authors, without undue reservation.

## Supplementary material

Supplementary Figure 1 ROC curves of the remaining seven machine learning models. Logistic Regression, Neural Network, C5.0, Support Vector Machine, Naive Bayes, Elastic Net, and Decision Tree in the testing set. AUC, area under the curve; ROC, receiver operating characteristic.

Supplementary Figure 2 SHAP summary plot for the 19-feature XGBoost model. Feature importance ranking based on mean absolute SHAP values from LASSO-selected features. Red indicates high feature values; blue indicates low values. HN, hydronephrosis; SHAP, SHapley Additive exPlanations.

Supplementary Figure 3 Decision curve analysis of XGBoost models with different feature sets. Models with 7 or more features demonstrated superior net benefit compared to “treat all” and “treat none” strategies across threshold probabilities from 5% to 95%.
