# Supplementary material for: An explainable machine learning model for predicting bladder tumor aecurrence risk
Source: Front Oncol. 2026 Jan 29;16:1728056. doi: 10.3389/fonc.2026.1728056 (PMC12896212; doi:10.3389/fonc.2026.1728056)
Supplement: Supplementary file 1 [file Supplementaryfile1.docx]

| Supplementary Table 1. Demographic and Clinical Characteristics of Patients | | | | | |
| --- | --- | --- | --- | --- | --- |
| Characteristics | Subgroups | All patients (n=504) | recurrence (n=90) | non-recurrence  (n=414) | P value* |
| gender | Male | 391 (77.6%) | 69 (76.7%) | 322 (77.8%) | 0.929 |
|  | Female | 113 (22.4%) | 21 (23.3%) | 92 (22.2%) |  |
| Marital.Status | Single | 14 (2.8%) | 4 (4.4%) | 10 (2.4%) | 0.499 |
|  | Married | 484 (96.0%) | 86 (95.6%) | 398 (96.1%) |  |
|  | Divorced | 1 (0.2%) | 0 (0.0%) | 1 (0.2%) |  |
|  | Widowed | 5 (1.0%) | 0 (0.0%) | 5 (1.2%) |  |
| Education.Level | Elementary | 290 (57.5%) | 64 (71.1%) | 226 (54.6%) | 0.034* |
|  | Middle | 124 (24.6%) | 12 (13.3%) | 112 (27.1%) |  |
|  | High | 53 (10.5%) | 8 (8.9%) | 45 (10.9%) |  |
|  | College | 2 (0.4%) | 0 (0.0%) | 2 (0.5%) |  |
|  | University | 35 (6.9%) | 6 (6.7%) | 29 (7.0%) |  |
| Registration | Rural | 249 (49.4%) | 47 (52.2%) | 202 (48.8%) | 0.636 |
|  | Urban | 255 (50.6%) | 43 (47.8%) | 212 (51.2%) |  |
| Smoking | No | 359 (71.2%) | 25 (27.8%) | 334 (80.7%) | <0.001*** |
|  | Yes | 145 (28.8%) | 65 (72.2%) | 80 (19.3%) |  |
| Alcohol | No | 400 (79.4%) | 62 (68.9%) | 338 (81.6%) | 0.010* |
|  | Yes | 104 (20.6%) | 28 (31.1%) | 76 (18.4%) |  |
| Hx.of.Bladder.Stones | No | 446 (88.5%) | 45 (50.0%) | 401 (96.9%) | <0.001*** |
|  | Yes | 58 (11.5%) | 45 (50.0%) | 13 (3.1%) |  |
| Hx.of.Chronic.UTI | No | 390 (77.4%) | 56 (62.2%) | 334 (80.7%) | <0.001*** |
|  | Yes | 114 (22.6%) | 34 (37.8%) | 80 (19.3%) |  |
| Hypertension | No | 251 (49.8%) | 33 (36.7%) | 218 (52.7%) | 0.008** |
|  | Yes | 253 (50.2%) | 57 (63.3%) | 196 (47.3%) |  |
| Diabetes | No | 398 (79.0%) | 47 (52.2%) | 351 (84.8%) | <0.001*** |
|  | Yes | 106 (21.0%) | 43 (47.8%) | 63 (15.2%) |  |
| Coronary.Heart.Disease | No | 394(78.2%) | 59 (65.6%) | 335 (80.9%) | 0.002** |
|  | Yes | 110 (21.8%) | 31 (34.4%) | 79 (19.1%) |  |
| Chronic.Kidney.Disease | No | 476 (94.4%) | 86 (95.6%) | 390 (94.2%) | 0.291 |
|  | Yes | 28 (5.6%) | 4 (4.4%) | 24 (5.8%) |  |
| Cerebrovascular.Disease | No | 472 (93.7%) | 87 (96.7%) | 385 (93.0%) | 0.599 |
|  | Yes | 32 (6.3%) | 3 (4.4%) | 29 (7.0%) |  |
| Prostate.Disease | No | 370 (73.4%) | 74 (82.2%) | 296 (71.5%) | 0.051 |
|  | Yes | 134 (26.6%) | 16 (17.8%) | 118 (28.5%) |  |
| Hematuria | No | 312 (61.9%) | 62 (68.9%) | 250 (60.4%) | 0.166 |
|  | Yes | 192 (38.1%) | 28 (31.1%) | 164 (39.6%) |  |
| Urine.RBC | Negative | 181 (35.9%) | 21 (23.3%) | 160 (38.6%) | 0.009** |
|  | Positive | 323 (64.1%) | 69 (76.7%) | 254 (61.4%) |  |
| Urine.WBC | Negative | 237 (47.0%) | 34 (37.8%) | 203 (49%) | 0.068 |
|  | Positive | 267 (53.0%) | 56 (62.2%) | 211 (51.0%) |  |
| Multiple.Tumors | Single | 306 (60.7%) | 64 (71.1%) | 280 (67.6%) | <0.001*** |
|  | Multiple | 198 (39.3%) | 41 (60.3%) | 134 (32.4%) |  |
| Extravesical.Invasion.Signs | No | 386 (76.6%) | 33 (36.7%) | 353 (85.3%) | <0.001*** |
|  | Yes | 118 (23.4%) | 57 (63.3%) | 61 (14.7%) |  |
| HN | No | 441 (87.5%) | 62 (68.9%) | 379 (91.5%) | <0.001*** |
|  | Yes | 63 (12.5%) | 28 (31.1%) | 35 (8.5%) |  |
| Base.Type | Narrow-based | 182 (36.1%) | 58 (64.4%) | 124 (30.0%) | <0.001*** |
|  | Broad-based | 322 (63.9%) | 32 (35.6%) | 290 (70.0%) |  |
| Morphology | Papillary | 316 (62.7%) | 20 (22.2%) | 296 (71.5%) | <0.001*** |
|  | Solid | 128 (25.4%) | 31 (34.4%) | 97 (23.4%) |  |
|  | Mixed | 60 (11.9%) | 39 (43.3%) | 21 (5.1%) |  |
| Trigone | No | 323 (64.1%) | 40 (44.4%) | 283 (68.4%) | <0.001*** |
|  | Yes | 181 (35.9%) | 50 (55.6%) | 131 (31.6%) |  |
| Rt.Lateral.Wall | No | 318 (63.1%) | 49 (54.4%) | 269 (65.0%) | 0.079 |
|  | Yes | 186 (36.9%) | 41 (45.6%) | 145 (35.0%) |  |
| Lt.Lateral.Wall | No | 336 (66.7%) | 54 (60.0%) | 282 (68.1%) | 0.175 |
|  | Yes | 168 (33.3%) | 36 (40.0%) | 132 (31.9%) |  |
| Ant.Wall | No | 382 (75.8%) | 63 (70.0%) | 319 (77.1%) | 0.201 |
|  | Yes | 122 (24.2%) | 27 (30.0%) | 95 (22.9%) |  |
| Post.Wall | No | 440 (87.3%) | 72 (80.0%) | 368 (88.9%) | 0.034* |
|  | Yes | 64 (12.7%) | 18 (20.0%) | 46 (11.1%) |  |
| Dome | No | 386 (76.6%) | 39 (43.3%) | 347 (83.8%) | <0.001*** |
|  | Yes | 118 (23.4%) | 51 (56.7%) | 67 (16.2%) |  |
| Neck | No | 438 (86.9%) | 77 (85.6%) | 361 (87.2%) | 0.982 |
|  | Yes | 66 (13.1%) | 13 (14.4%) | 53 (12.8%) |  |
| Anesthesia.Type | GA | 471 (93.5%) | 85 (94.4%) | 386 (93.2%) | 0.805 |
|  | SA | 33 (6.5%) | 5 (5.6%) | 28 (6.8%) |  |
| Bladder.Instillation | No | 227 (45.0%) | 36 (40.0%) | 191 (46.1%) | 0.345 |
|  | Yes | 277 (55.0%) | 54 (60.0%) | 223 (53.9%) |  |
| Immunotherapy | No | 437 (86.7%) | 78 (86.7%) | 359 (86.7%) | 1.000 |
|  | Yes | 67 (13.3%) | 12 (13.3%) | 55 (13.3%) |  |
| Age(years) |  | 72.0(65.0, 79.0) | 74.5 (70.0, 82.0) | 71.0 (64.3, 78.0) | 0.001** |
| BMI(kg/m²) |  | 23.8 (21.4, 26.1) | 25.6 (24.3, 28.1) | 23.4 (20.9, 25.5) | <0.001*** |
| Urine.Specific.Gravity |  | 1.02 (1.01, 1.02) | 1.02 (1.01, 1.02) | 1.02 (1.01, 1.02) | 0.842 |
| Urine.pH |  | 6.50 (6.00, 6.62) | 6.00 (6.00, 6.50) | 6.50 (6.00, 7.00) | 0.043* |
| Hb(g/L) |  | 138 (126, 148) | 138 (132, 148) | 137 (125, 148) | 0.397 |
| RBC(×10¹²/L) |  | 4.47 (4.06, 4.78) | 4.50 (4.24, 4.74) | 4.45 (4.04, 4.80) | 0.479 |
| Cr(mmol/L) |  | 73.3 (61.7, 87.3) | 74.4 (64.7, 90.0) | 73.1 (60.7, 87.3) | 0.407 |
| BUN(mmol/L) |  | 5.75 (4.83, 7.16) | 5.74 (4.81, 7.12) | 5.87 (5.25, 7.45) | 0.112 |
| eGFR(ml/min/1.73m²) |  | 86.8 (72.8, 94.5) | 83.8 (61.9, 92.6) | 87.4 (74.4, 95.0) | 0.136 |
| ALT(U/L) |  | 18.0 (13.0, 25.0) | 18.0 (13.0, 26.0) | 18.0 (13.0, 22.3) | 0.925 |
| AST(U/L) |  | 23.0 (19.0, 28.0) | 23.0 (20.8, 30.0) | 22.5 (19.0, 27.0) | 0.017* |
| Alb(g/L) |  | 43.3 (39.9, 45.6) | 44.2 (41.5, 46.2) | 43.1 (39.7, 45.5) | 0.009** |
| TBIL(μmol/L) |  | 12.0 (9.0, 16.0) | 11.0 (9.0, 16.0) | 12.0 (8.0, 16.5) | 0.689 |
| Na(mmol/L) |  | 141 (139, 142) | 142 (140, 143) | 141 (139, 142) | 0.003** |
| K(mmol/L) |  | 4.09 (3.86, 4.35) | 4.08 (3.85, 4.34) | 4.16 (3.92, 4.38) | 0.193 |
| Tumor.Number |  | 2.00 (1.00, 3.00) | 3.00 (2.00, 4.00) | 1.00 (1.00, 2.00) | <0.001*** |
| Max.Tumor.Diameter |  | 1.00 (0.80, 2.50) | 2.75 (2.00, 3.48) | 1.00 (0.80, 2.00) | <0.001*** |
| Operation.Time(min) |  | 33.0 (23.0, 50.3) | 36.0(24.8, 48.0) | 32.0 (22.0, 51.0) | 0.497 |
| Catheter.Duration(day) |  | 2.00 (1.00, 4.00) | 2.00 (1.00, 5.00) | 2.00 (1.00, 3.25) | 0.153 |
| Length.of.Stay |  | 5.00 (3.00, 9.00) | 5.50 (4.00, 9.00) | 5.00 (3.00, 8.25) | 0.630 |

Notes: BMI, body mass index. Hb, hemoglobin. RBC, red blood cell. WBC, white blood cell. Cr, creatinine. BUN, blood urea nitrogen. eGFR, estimated glomerular filtration rate. ALT, alanine aminotransferase. AST, aspartate aminotransferase. Alb, albumin. TBIL, total bilirubin. Na, sodium. K, potassium. HN, hydronephrosis.
