# Supplementary figures and images for: An explainable machine learning model for predicting bladder tumor aecurrence risk
Source: Front Oncol. 2026 Jan 29;16:1728056. doi: 10.3389/fonc.2026.1728056 (PMC12896212; doi:10.3389/fonc.2026.1728056)

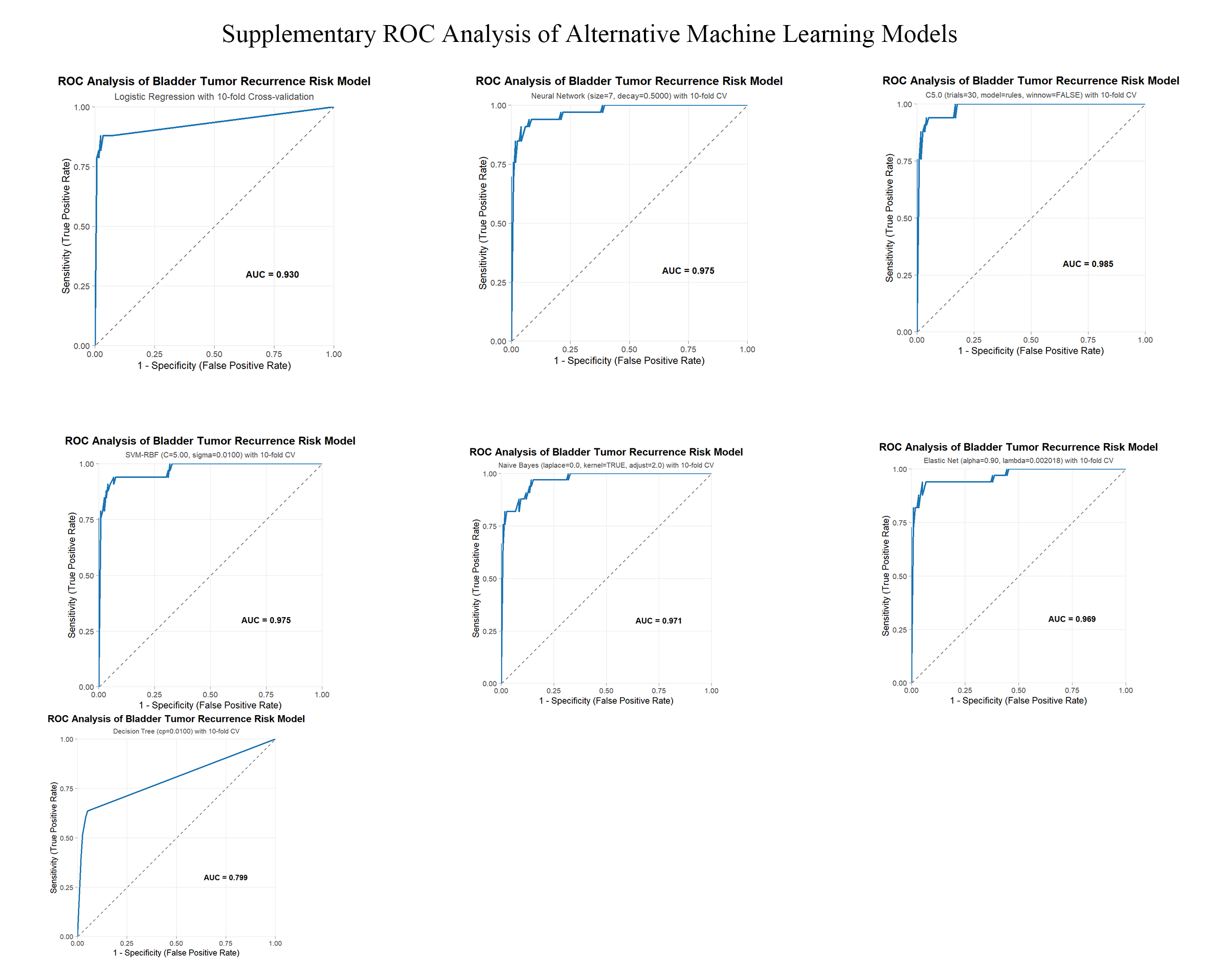

Supplement: Supplementary Figure 1 — ROC curves of the remaining seven machine learning models. Logistic Regression, Neural Network, C5.0, Support Vector Machine, Naive Bayes, Elastic Net, and Decision Tree in the testing set. AUC, area under the curve; ROC, receiver operating characteristic. [file Image1.tif]

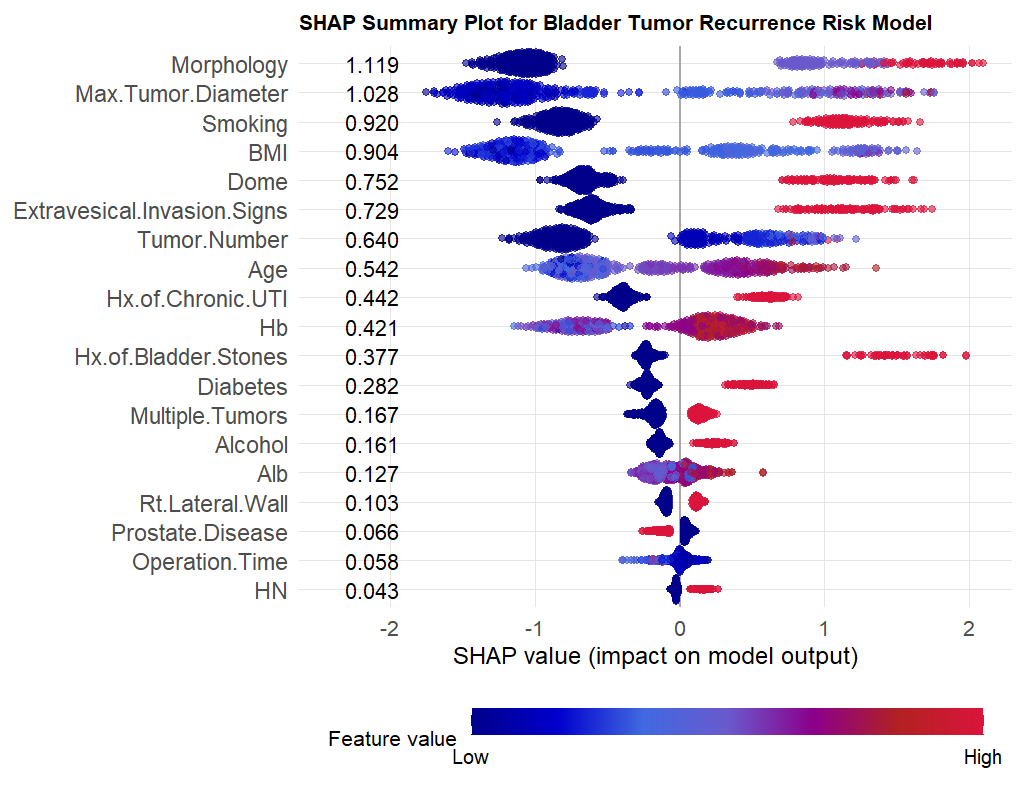

Supplement: Supplementary Figure 2 — SHAP summary plot for the 19-feature XGBoost model. Feature importance ranking based on mean absolute SHAP values from LASSO-selected features. Red indicates high feature values; blue indicates low values. HN, hydronephrosis; SHAP, SHapley Additive exPlanations. [file Image2.tiff]

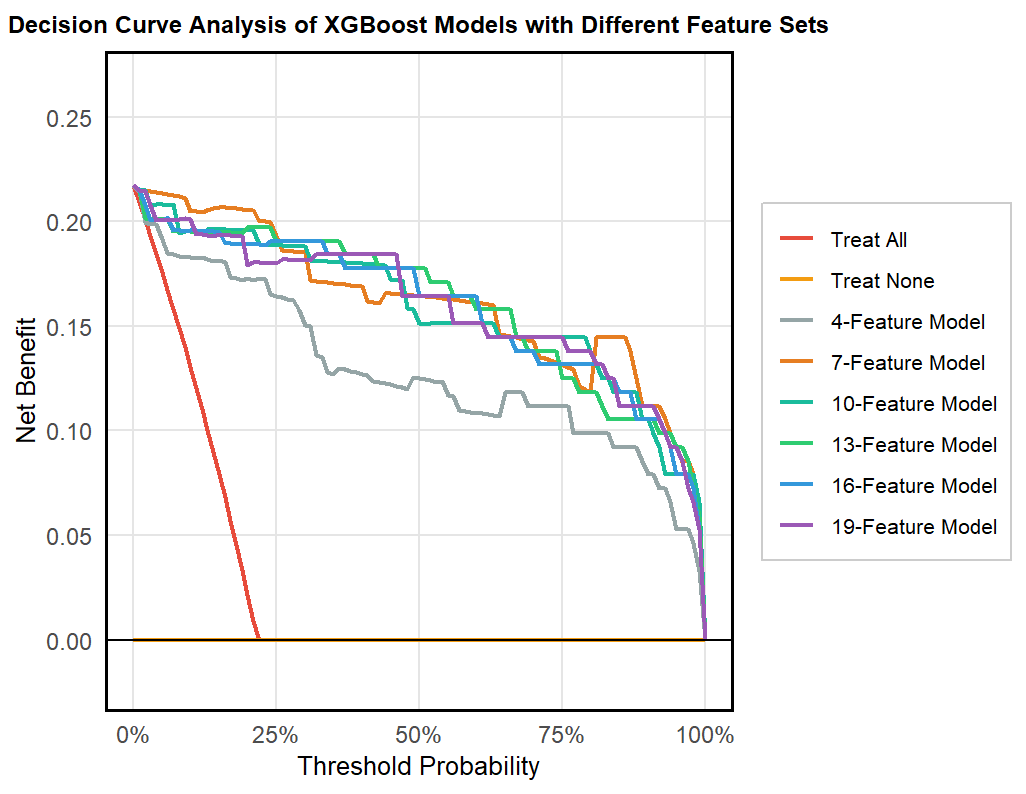

Supplement: Supplementary Figure 3 — Decision curve analysis of XGBoost models with different feature sets. Models with 7 or more features demonstrated superior net benefit compared to “treat all” and “treat none” strategies across threshold probabilities from 5% to 95%. [file Image3.tiff]
